# Analytical performance validation of aPROMISE platform for prostate tumor burden, index and dominant tumor assessment with 18F-DCFPyL PET/CT. A pilot study

**DOI:** 10.1038/s41598-024-53683-z

**Published:** 2024-02-06

**Authors:** Ana María García Vicente, Cristina Lucas Lucas, Julián Pérez-Beteta, Pablo Borrelli, Laura García Zoghby, Mariano Amo-Salas, Ángel María Soriano Castrejón

**Affiliations:** 1 Nuclear Medicine Department, Complejo Hospitalario Universitario de Toledo, Avda. Rio Guadiana s/n, 45007 Toledo, Spain; 2 Nuclear Medicine Department, University General Hospital, Ciudad Real, Spain; 3 Mathematical Oncology Laboratory (MOLab), Castilla-La Mancha University, Ciudad Real, Spain; 4 Department of Mathematics, Castilla-La Mancha University, Ciudad Real, Spain; 5 Department of Clinical Physiology, Region Västra Götaland, Sahlgrenska University Hospital, Gothenburg, Sweden

**Keywords:** Molecular medicine, Machine learning

## Abstract

To validate the performance of automated Prostate Cancer Molecular Imaging Standardized Evaluation (aPROMISE) in quantifying total prostate disease burden with 18F-DCFPyL PET/CT and to evaluate the interobserver and histopathologic concordance in the establishment of dominant and index tumor. Patients with a recent diagnosis of intermediate/high-risk prostate cancer underwent 18F-DCFPyL-PET/CT for staging purpose. In positive-18F-DCFPyL-PET/CT scans, automated prostate tumor segmentation was performed using aPROMISE software and compared to an in-house semiautomatic-manual guided segmentation procedure. SUV and volume related variables were obtained with two softwares. A blinded evaluation of dominant tumor (DT) and index tumor (IT) location was assessed by both groups of observers. In histopathological analysis, Gleason, International Society of Urological Pathology (ISUP) group, DT and IT location were obtained. We compared all the obtained variables by both software packages using intraclass correlation coefficient (ICC) and Cohen’s kappa coefficient (k) for the concordance analysis. Fifty-four patients with a positive ^18^F-DCFPyL PET/CT were evaluated. The ICC for the SUVmax, SUVpeak, SUVmean, tumor volume (TV) and total lesion activity (TLA) was: 1, 0.833, 0.615, 0.494 and 0.950, respectively (p < 0.001 in all cases). For DT and IT detection, a high agreement was observed between both softwares (k = 0.733; p < 0.001 and k = 0.812; p < 0.001, respectively) although the concordances with histopathology were moderate (p < 0001). The analytical validation of aPROMISE showed a good performance for the SUVmax, TLA, DT and IT definition in comparison to our in-house method, although the concordance was moderate with histopathology for DT and IT.

## Introduction

Positron emission tomography (PET) with ligands against prostate specific membrane antigen (PSMA) seems as an effective noninvasive method for prostate cancer (pCa) characterization and localization^1,2^.

Expression of PSMA, addressed by PSMA PET, increases on tumors with higher Gleason score (GS)^3,4^. The most aggressive part of the prostatic tumor, knowing as dominant tumor (DT), usually based on histology, can be assessed by PET, although with a scarcely reported use^5,6^. In addition, the percentage of tumor present or total tumor length on core biopsies, known as index tumor (IT), has been reported as a stronger prognostic information about overall survival than does patient age, serum prostate specific antigen (PSA) level, or even GS^7^.

In morphological imaging, manual contouring of pCa is considered as the gold standard in the radiotherapy approach^8^. Moreover, an accurate contouring of the intraprostatic gross tumor volume (GTV) is mandatory for successful fusion-biopsy guidance and focal therapy approaches^9,10^. However, PSMA PET–based GTV definition underlies a substantial interobserver variability and expenditure of time and in addition, actually, there is not a validated proposed contouring technique^11^.

The objective of the present work, was to validate the performance of the automated Prostate Cancer Molecular Imaging Standardized Evaluation (aPROMISE) software in quantifying total prostate disease burden in patients with intermediate and high-risk pCa who underwent 18F-DCFPyL [2-(3-(1-carboxy-5-[(6-[18F]fluoro-pyridine-3-carbonyl)-amino]-pentyl)-ureido)-pentanedioic acid] for staging purposes and to evaluate the interobserver concordance and with the histopathologic analysis in the establishment of DT and IT.

## Material and methods

A retrospective analysis of a prospective dataset of consecutively included patients between March 2021 and June 2022 was approved by a reference Ethical Committee (registry code number: 2022-053).

All methods were performed following the relevant guidelines, local regulations and good clinical practice procedures.

### Patients

Patients with recent diagnosis of intermediate/high-risk pCa were consecutively derived for 18F-Fluorocholine-PET/computed tomography (CT) for staging purposes. The indication for 18F-DCFPyL-PET/CT was a previous negative/doubtful 18F-Fluorocholine-PET/CT for extraprostatic or compatible with oligometastatic disease. We established as oligometastatic disease the presence of ≤ 3 lesions affecting lymph node locations (in the pelvis and /or retroperitoneum) with possibility of one of them in bone.

18F-DCFPyL-PET/CT was performed within the context of compassionate use under the approval of the Spanish Agency of Medication and Health Care Products, after being approved by a multidisciplinary committee and with previous patient informed and signed consent.

Patients with androgen deprivation therapy initiation previous to any of PET/CT or lost in the follow-up were excluded.

Prostate specific antigen level and TNM based on CT and/or pelvic magnetic resonance imaging (MRI) were collected to obtain the D’Amico risk category. High-risk patients were defined if they met at least one of the following criteria: International Society of Urological Pathology (ISUP) grade group 4/5 or PSA ≥ 20 ng/mL or clinical tumor stage T3. Intermediate-risk was defined if ISUP grade group 2/3 or PSA 10-20 ng/mL or clinical tumor stage T2b-c^12^.

### ^18^F-DCFPyL PET/CT acquisition and analysis

18F-DCFPyL PET/CTs was performed in a hybrid PET/CT scanner (Discovery 5R/IQ, General Electric), in 3D acquisition mode for 2 min per bed position, 120 min after intravenous administration of an activity of 2–4 MBq/kg. Diuretic was administered 1 h after radiotracer injection. Low dose CT (120 kV, 80 mA) without contrast was performed for attenuation correction and as anatomical map.

Prostate axial slices of 18F-DCFPyL PET/CT were visually assessed independently by two experienced observers belonging to two investigational groups. In PSMA-positive studies, automated prostate tumor segmentation was performed using aPROMISE software^13^ and compared to the scientific software package Matlab (R2021b, MathWorks, Natick, Mass) using an in-house semiautomatic-manual guided segmentation procedure developed by the Mathematical Oncology Laboratory group (MOLab) based on a gradient algorithm detailed in previous publications^14,15^. Two nuclear medicine physicians revised all the procedures.

aPROMISE (version 2.2.1), is a class II software (web application) developed by EXINI Diagnostics AB (Lund, Sweden) to standardize and quantify PSMA-positive findings in imaging of patients with pCa. Deep learning is used to automatically analyse the CT image to segment anatomic regions, including individual bones, and soft-tissue organs such as the prostate. The anatomical references are used to provide staging of the disease regarding extension of the prostatic tumor as the involvement of locoregional lymph nodes and distant metastases. Afterwards the corresponding PET images are analysed to detect so called “target” lesions (lesions showing pathologically increased PSMA-uptake). Currently the aPROMISE software uses the PROMISE criteria as a standard guideline for PSMA assessment^16^. Results are obtained by merging molecular imaging lesion information with the corresponding anatomical location and shown as miPSMA index per-lesion and aggregated per-region/per-typ^13^.

After tumor segmentation, a visual check was performed to exclude physiological urinary activity from the segmentation. Standardized uptake value (SUV) variables [SUVmax, SUVpeak, SUVmean] and volume-based variables as PSMA tumor volume (TV) and total lesion activity (TLA) were obtained.

Two observers performed a blinded evaluation, of DT and IT location. DT was considered as the prostate lobe with the highest SUVmax and IT as the prostate lobe with the biggest molecular TV.

### Histopathological analysis

The location of pCa attending to lobes (right, left or both) and Gleason group, considering the higher GS of the total core biopsies, was established.

The ISUP grade group (1 to 5) was obtained by histological analysis of multiple biopsy specimens of prostate gland^17^.

The lobe of the IT location was considered as the lobe with the greatest number of positive core biopsies. The average (mean) of percentage of tumor involvement on core biopsies was obtained summing all and dividing by the number of the positive ones. The lobe with the DT was the lobe with the highest GS on positive core biopsies. In addition, perineural invasion and angiolymphatic invasion per lobes was obtained.

### Statistical analysis

For the statistical analysis, SPSS software (v. 28) was used. Descriptive analysis considered mean and standard deviation (SD) for quantitative variables whereas absolute and relative frequencies were considered for qualitative variables. In the concordance analysis, the Cohen’s kappa coefficient (k) was used to study the interobserver concordance with respect to the DT and IT, and the concordance between each observer and the final histopathologic result on prostate biopsy was assessed. The results were classified as poor (< 0.20), weak (0.21–0.40), moderate (0.41–0.60), good (0.61–0.80) and very good (0.81–1.00). The interobserver concordance with respect to the quantitative variables (SUVmax, SUVpeak, SUVmean, TV and TLA) was studied using the intraclass correlation coefficient (ICC).

Moreover, we compared the means of the variables obtained with the aPROMISE and with our in-house MOLab assisted packages in the total sample of patients and attending to ISUP (group A), risk categories (group B) and perineural invasion (group C) using paired sample T-test, for the comparison between packages, and ANOVA analysis, for the comparison in each package. Statistically significant differences were considered when p < 0.05.

### Ethics approval

Study was approved by a reference Ethical Committee (Gerencia de Atención integrada de Albacete). Registry code number: 2022-053. All the authors have participated in the writing and revision of this article and take public responsibility for its content. The present publication is approved by all authors and by the responsible authorities where the work was carried out. All the authors confirm the fact that the article is not under consideration for publication elsewhere.

### Consent for participating and for publication

Patients signed an informed consent to participate and to use their anonymous data for analysis and publication of results.

## Results

Fifty-eight patients were evaluated although 54 positive on ^18^F-DCFPyL PET/CT were finally included in the analysis. 46/54 (85.2%) were high-risk pCa and 23/53 (43.4%) ISUP 4 or 5 tumors. The percentage of tumor involvement of the global sample of patients ranged from 4 to 100%. Table 1 summarizes all the tumor characteristics.

**Table 1:** Disease´s characteristics.

Characteristics	Value
Age (years) mean ± SD	68.37 ± 7.69
ISUP grade group*	
1	6 (11.3%)
2	16 (30.2%)
3	8 (15.1%)
4	12 (22.6%)
5	11 (20.8%)
Risk	
Intermediate	8 (14.8%)
High	46 (85.2%)
Angiolymphatic invasion (yes/no)	1 (1.9%)/53 (98.1%)
Perineural invasion (yes/no)	18 (33.4%)/36 (66.6%)

*ISUP* International Society of Urological Pathology.

*One missing data.

Dominant tumor was located in right lobe for the observers of aPROMISE and MOLab in 31 and 32 patients, respectively with a high agreement (k = 0.73; p < 0.001) and the IT in 32 and 29 patients, respectively with a high agreement (k = 0.81; p < 0.001). However, the concordances between observers and histopathology were moderate (p < 0001). Table 2*.*

**Table 2:** Distribution of dominant and index tumor attending to the two software and their comparison with histopathologic distribution.

A					
		DT Lobe MOLab			Total
		Right	Left		
DT lobe aPROMISE	Right	28	3		31
	Left	4	19		23
Total		32	22		54

B					
		IT Lobe MOLab			Total
		Right	Left		
IT lobe aPROMISE	Right	28	4		32
	Left	1	21		22
Total		29	25		54

C					
		Histopathologic DT			
		Right	Left	Both	
DT lobe aPROMISE	Right	23	3	5	31
	Left	2	17	4	23
Total		25	20	9	54

D					
		Histopathologic IT			Total
		Right	Left	Both	
IT lobe aPROMISE	Right	23	6	3	32
	Left	4	17	1	22
Total		27	23	4	54

E					
		Histopathologic DT			Total
		Right	Left	Both	
DT lobe MOLab	Right	25	4	3	32
	Left	0	16	6	22
Total		25	20	9	54

F					
		Histopathologic IT			Total
		Right	Left	Both	
IT lobe MOLab	Right	22	6	1	29
	Left	5	17	3	25
Total		27	23	4	54

The kappa values of the different concordances were: A: K = 0.733; B: K = 0.812; C: K = 0.550; D: K = 0.511; E: K = 0.581 and F: K = 0.480 with p < 0.001 in all the cases.

Regarding DT and IT localization, the concordance between aPROMISE and MOLab was high (k = 0.89, p < 0.001 for both).

In prostate tumor, the mean ± SD of SUVmax, SUVpeak, SUVmean, TV and TLA for MOLab vs aPROMISE were: 34.31 ± 32.45 vs. 34.53 ± 32.65, 18.75 ± 17.39 vs. 14.02 ± 12.46, 11.20 ± 7.31 vs. 6.09 ± 4.67, 10.27 ± 10.85 vs. 24.18 ± 16.04 and 149.65 ± 275.01 vs. 182.21 ± 340.51. ICC for the previous semiquantitative variables obtained in both packages are detailed on Table 3.

**Table 3:** Global SUV and volume based PET variables and their intraclass correlation coefficient.

Characteristics		Mean ± SD	Maximum and minimum values	ICC	p
SUVmax	MOLab	34.31 ± 32.45	4.80; 174.37	1	< 0.001
	aPROMISE	34.53 ± 32.65	4.83; 175.47		
SUVmean	MOLab	11.2 ± 7.32	3.05; 45.02	0.615	< 0.001
	aPROMISE	6.09 ± 4.68	2.61; 33.62		
SUVpeak	MOLab	18.76 ± 17.39	3.96; 109.16	0.833	< 0.001
	aPROMISE	14.02 ± 12,46	2.94; 67.28		
TV	MOLab	10.26 ± 10.85	0.30; 56.07	0.495	< 0.001
	aPROMISE	24.19 ± 16.04	2.38 ; 74.53		
TLA	MOLab	149.65 ± 275.02	1.17; 1894.36	0.950	< 0.001
	aPROMISE	182.21 ± 340.51	6.23; 2505.82		

**Figure 1:**
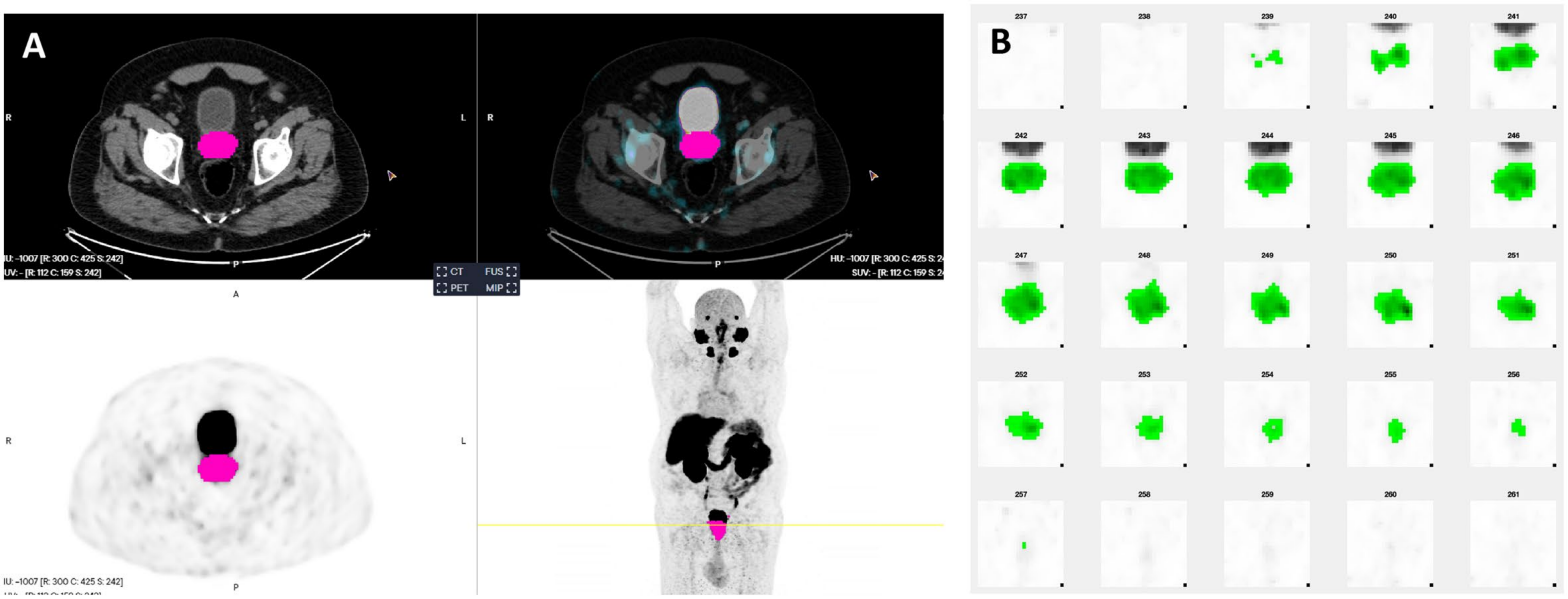
(**A**) Axial slices of segmented tumor on aPROMISE software. (**B**) Segmented tumor using MOLab software of an ISUP 5 adenocarcinoma involving both lobes. SUVmax, SUVmean, SUVpeak, TV and TLA by aPROMISE of 30.10, 6.78, 14.63, 69.23 and 468.97, respectively. The correspondent values for MOLab were of 29.98, 10.15, 15.21, 56.07 and 569.75, respectively.

**Figure 2:**
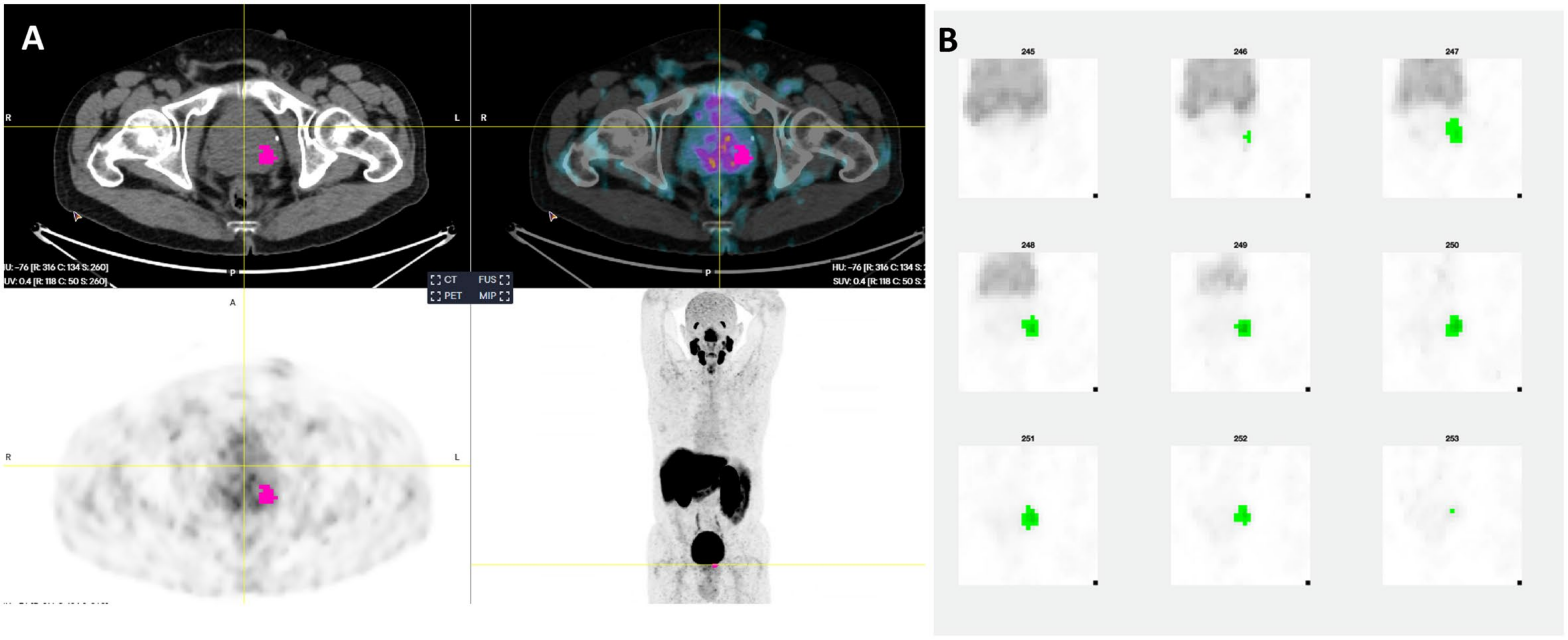
(**A**) Axial slices of segmented tumor on aPROMISE software. (**B**) Segmented tumor using MOLab software. Dominant and index tumor are located in left lobe. Histopathology: Gleason (3 + 3) adenocarcinoma in both lobes involving 2/6 cores. Dominant and index tumor in both lobes.

**Figure 3:**
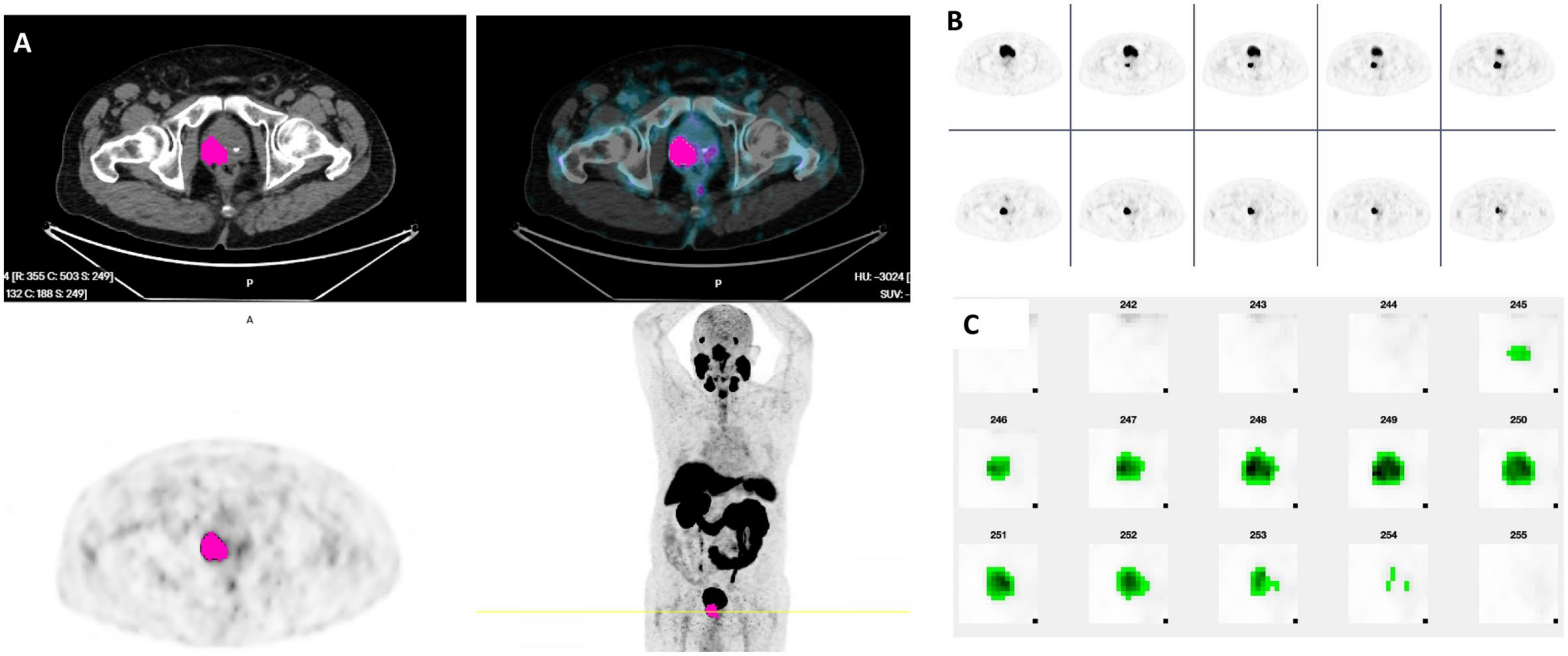
(**A**) Axial slices of segmented tumor on aPROMISE software. (**B**) Consecutive axial slices on PET. (**C**) Segmented tumor using MOLab software. Dominant and index tumor are located in right lobe. Histopathology: right lobe: Gleason (4 + 3) adenocarcinoma, 8/8 cores and left lobe: Gleason (3 + 4) adenocarcinoma, 5/9 cores. Dominant and index tumor in right lobe.

For any individual segmentation package, no significant differences of SUV and volume-based variables with the different ISUP grade groups and risk-categories were observed (Table 4). However, significant differences were detected between both segmentation packages attending to the different ISUP group grades, risk-categories and perineural invasion groups, being semiquantitative parameters obtained by aPROMISE bigger than MOLab, except for the TLA and ISUP grade groups. On the contrary, MOLab had superior values of SUVmean and SUVpeak with respect to aPROMISE (Table 5). Some case examples are showed in Figs. 1, 2 and 3.

**Table 4:** SUV and volume based parameters (mean ± SD) for any individual software package.

Group A							
ISUP							
		1	2	3	4	5	p
SUVmax	MOLab	32.21 ± 22.64	33.96 ± 36.40	47.62 ± 54.34	32.84 ± 25.53	27.28 ± 32.76	0.784
	aPROMISE	32.41 ± 22.78	34.18 ± 36.63	47.94 ± 54.67	33.94 ± 25.69	27.93 ± 19.01	0.783
SUVmean	MOLab	11.61 ± 6.21	10.33 ± 9.64	12.69 ± 8.15	11.60 ± 6.48	10.44 ± 5.25	0.954
	aPROMISE	5.87 ± 2.72	6.26 ± 7.41	6.90 ± 4.33	5.71 ± 2.44	5.62 ± 2.98	0.980
SUVpeak	MOLab	19.55 ± 14.90	19.61 ± 25.02	21.67 ± 17.54	18.83 ± 14.54	14.29 ± 8.36	0.920
	aPROMISE	17.72 ± 14.39	12.98 ± 15.60	16.04 ± 12.95	13.60 ± 11.18	11.90 ± 8.79	0.895
TV	MOLab	6.73 ± 5.20	9.81 ± 9.70	8.62 ± 4.69	8.20 ± 7.30	15.84 ± 18.54	0.400
	aPROMISE	16.86 ± 10.17	24.93 ± 16.1	22.17 ± 9.28	22.36 ± 18.79	29.28 ± 19.65	0.633
TLA	MOLab	96.89 ± 111.24	182.34 ± 459.92	136.24 ± 151.52	116.92 ± 188.68	170.26 ± 188.68	0.957
	aPROMISE	115.55 ± 97.05	248.95 ± 604.15	153.88 ± 119.90	143.66 ± 128.35	173.51 ± 142.97	0.911

Group B							
Risk classification							
		Intermediate (n = 8)			High (n = 46)		p
SUVmax	MOLab	26.97 ± 17.25			35.40 ± 34.13		0.728
	aPROMISE	27.14 ± 17.35			35.63 ± 34.34		0.726
SUVmean	MOLab	8.51 ± 3.64			11.60 ± 7.66		0.562
	aPROMISE	4.92 ± 1.65			6.26 ± 4.96		0.652
SUVpeak	MOLab	15.10 ± 10.26			19.3 ± 18.23		0.787
	aPROMISE	11.44 ± 7.83			14.41 ± 13.02		0.867
TV	MOLab	8.1 ± 5.64			10.59 ± 11.44		0.787
	aPROMISE	21.62 ± 10.49			24.57 ± 16.76		0.918
TLA	MOLab	80.97 ± 76.35			159.88 ± 292.51		0.652
	aPROMISE	113.83 ± 73.54			192.40 ± 363.41		0.748

Group C							
Angiolymphatic invasion							
		Yes (n = 18)			No (n = 36)		p
SUVmax	MOLab	27.88 ± 21.19			37.52 ± 36.67		0.322
	aPROMISE	28.06 ± 21.32			37.76 ± 36.89		0.322
SUVmean	MOLab	10.11 ± 5.84			11.75 ± 7.97		0.509
	aPROMISE	5.1 ± 2.21			6.59 ± 5.47		0.322
SUVpeak	MOLab	16.03 ± 12.18			20.12 ± 19.49		0.474
	aPROMISE	10.99 ± 8.99			15.54 ± 13.74		0.137
TV	MOLab	13.11 ± 15			8.85 ± 7.93		0.441
	aPROMISE	29.71 ± 19.86			21.42 ± 13.21		0.196
TLA	MOLab	142.67 ± 163.26			153.14 ± 318.67		0.700
	aPROMISE	166.26 ± 134.86			190.19 ± 409.1		0.545

**Table 5:** Differences between MOLab and aPROMISE software regarding histology, risk classification and angiolymphatic invasion.

ISUP	T value and p (MOLab vs aPROMISE)				
	SUVmax	SUVmean	SUVpeak	MTV	TLA
1	− 3.49; 0.018	3.82; 0.013	0.79; 0.472	− 4.30; 0.008	− 1.30; 0.251
2	− 3.77; 0.002	6.31; < 0.001	2.64; 0.018	− 6.61; < 0.001	− 1.8; 0.092
3	− 2.71; 0.030	3.20; 0.015	1.73; 0.128	− 4.94; 0.002	− 1.15; 0.288
4	− 4.46; < 0.001	4.50; < 0.001	2.47; 0.031	− 3.31: 0.007	− 1.59; 0.141
5	− 4.87; < 0.001	5.55; < 0.001	3.11; 0.011	− 5.95; < 0.001	− 0.16; 0.878
Risk					
Intermediate	− 4.36; 0.005	4.13; 0.006	2.81; 0.031	− 4.10; 0.006	− 2.77; 0.033
High	− 7.26; < 0.001	9.80; < 0.001	4.13; < 0.001	− 9.64; < 0.001	− 2.23; 0.031
Angiolymphatic invasion					
Yes	− 5.67; < 0.001	5.34; < 0.001	3.61; 0.002	− 5.67; < 0.001	− 1.68; 0.111
No	− 6.30; < 0.001	8.91; < 0.001	3.25; 0.003	− 9.55; < 0.001	− 2.10; 0.046

## Discussion

An improvement on the detection of the more active tumor, as a representation of the DT, using PSMA PET, can provide a higher possibility of lesion detection on guided biopsies, based on SUV values of PSMA expression are usually increased on tumors with higher GS^18–23^. However, in the present work no relations were found between semiquantitative PET variables with ISUP groups, similar to the reported by previous authors assessing SUVmax^24^. In addition, we used a novel approach of risk classification and although high-risk tumors had higher SUV and volume-based variables, no significant differences were found, probably based on the reduced sample of the different subgroups and the great dispersion of values. Paradoxically, perineural invasion was associated to lower SUV-based parameters and bigger TV although without significant differences.

In addition, significant differences were observed when paired comparison was obtained between both software, regarding to the ISUP, risk groups and perineural invasion. In fact, aPROMISE defined higher SUVmax and TV with respect to MOLab (Table 5). However, TLA assessed with two software did not show differences.

Approximately 5–10% pCa cells do not overexpress PSMA, that limits the PET detection even in intermediate-high risk pCa, explaining the false negative results in prostate tissue in our sample of patients^25–27^.

Previous authors have described higher uptake using PSMA ligands PET in the IT that exceeded the physiologic tracer uptake in normal prostate tissue (median SUVmax 12.5 vs 3.9)^28^. In the present work, the IT was defined as the area where the tumor showed its largest dimension^29^. In addition, no consensus exists regarding the definition of DT and IT in previous works and probably they are the same entity^30,31^. In the present work, the DT and IT were concordant in a majority of cases of our sample, supporting that usually the largest tumor yields the highest GS.

Lesion segmentation is the next step in order to select the TV using molecular imaging information derived from PET. Threshold-based contouring has been proposed although has intrinsic limitations^32,33^. Thus, efforts have been made to implement an automatic segmentation algorithm for improving the GTV delineation in PSMA PET images. This procedure has showed high concordance, with expert contours, and high sensitivities and specificities, in comparison with histology as reference^9,33^.

Finally, based on the restrictions of some conventional PET *radiomics* to provide acceptable diagnostic performance in differentiating pathological grade groups, we expect that perhaps other *radiomics,* offered by machine learning, would be more suitable as predictors^34,35^.

Regarding limitations, histopathologic analysis based on biopsy specimens, instead on surgical samples, could biases some ISUP results, explained by the lower detection rate of clinically significant pCa and a downgrading of GS to radical prostatectomy of the former concerning the latter^9,36–39^. In addition, the estimation of the percentage of tumor of core biopsies is a subjective process based on sometimes it is performed with independence of their dimensions. So, we avoided using the information of the percentage average (mean) of tumor involvement of core biopsies, as previous authors reported^7^. With respect to the strengths, this is the first reported experience of segmentation using two gradient-based semiautomatic procedures to obtain information of the most significant and extensive prostate tumor. Thus, despite the probably inherent limitations regarding the uncertainty in correlation of PSMA PET images, even with histopathologic slices^33^, prostate tumor segmentation seems feasible using semiautomatic algorithms.

## Conclusions

The analytical validation of aPROMISE showed a good performance for the SUVmax and TLA obtained after prostate tumor segmentation in comparison to our inhouse MOLab method in the global sample of patients. However, significant differences were detected between practically all the semiquantitative variables for the different ISUP groups and risk categories, facing up the highly procedure-dependence of the segmentation if these division is performed.

Total lesion activity was the unique method’s independent variable, postulating itself as the more robust and reproducible to be compared among software.

High agreement between two observers was achieved in the definition of DT and IT, using 18F-DCFPyL PET/CT, although there was moderate agreement with the histopathologic results, justifying the need to explore other *radiomics* and segmentation procedures.

## Data Availability

The datasets used and/or analysed during the current study available from the corresponding author on reasonable request.
